# Consolidated bioprocessing of transgenic switchgrass by an engineered and evolved *Clostridium thermocellum* strain

**DOI:** 10.1186/1754-6834-7-75

**Published:** 2014-05-22

**Authors:** Kelsey L Yee, Miguel Rodriguez Jr, Olivia A Thompson, Chunxiang Fu, Zeng-Yu Wang, Brian H Davison, Jonathan R Mielenz

**Affiliations:** 1 Biosciences Division, Oak Ridge National Laboratory, Oak Ridge, TN 37831-6341, USA; 2 BioEnergy Science Center, Oak Ridge National Laboratory, Oak Ridge, TN 37831-6037, USA; 3 Forage Improvement Division, The Samuel Roberts Noble Foundation, Ardmore, OK 73401, USA; 4 Current address: Qingdao Institute of Bioenergy and Bioprocess Technology, CAS, No.189 Songling Rd, Qingdao City, Shandong Province 266101, People’s Republic of China; 5 White Cliff Biosystems, Rockwood, TN 37854, USA

**Keywords:** Transgenic, Switchgrass, Metabolic engineering, *Clostridium thermocellum*, Consolidated bioprocessing, Feedstock, Cellulosic ethanol

## Abstract

**Background:**

Switchgrass is an abundant and dedicated bioenergy feedstock, however its inherent recalcitrance is one of the economic hurdles for producing biofuels. The downregulation of the caffeic acid O-methyl transferase (COMT) gene in the lignin pathway of switchgrass reduced lignin content and S/G ratio, and the transgenic lines showed improved fermentation yield with *Saccharomyces cerevisiae* and wild-type *Clostridium thermocellum* (ATCC 27405) in comparison to the wild-type switchgrass.

**Results:**

Here we examine the conversion and yield of the COMT transgenic and wild-type switchgrass lines with an engineered and evolved *C. thermocellum* (M1570) strain. The fermentation of the transgenic switchgrass by M1570 had superior conversion relative to the wild-type control switchgrass line with an increase in conversion of approximately 20% and ethanol being the primary product accounting for 90% of the total metabolites measured by HPLC analysis.

**Conclusions:**

The engineered and evolved *C. thermocellum* M1570 was found to respond to the apparent reduced recalcitrance of the COMT switchgrass with no substrate inhibition, producing more ethanol on the transgenic feedstock than the wild-type substrate. Since ethanol was the main fermentation metabolite produced by an engineered and evolved *C. thermocellum* strain, its ethanol yield on a transgenic switchgrass substrate (gram/gram (g/g) glucan liberated) is the highest produced thus far. This result indicates that the advantages of a modified feedstock can be combined with a modified consolidated bioprocessing microorganism as anticipated.

## Background

Lignocellulosic biomass is an abundant feedstock but its inherent recalcitrance towards conversion is one of the major economic hurdles to producing biofuels [1,2]. Lignin is a major component of plant cell walls and a contributor to recalcitrance by negatively impacting enzymatic hydrolysis of cellulose and hemicellulose to fermentable sugars [3,4]. Genetic engineering of feedstocks to reduce lignin content and/or improve composition has been shown to increase the accessibility of the cellulose and hemicellulose to enzymatic hydrolysis and as a result improve fermentation yield [5-11].

Although variation exists among these processes, the majority of lignocellulosic feedstocks to ethanol bioconversion schemes have four primary objectives: reduction of biomass particle size, biomass pretreatment, hydrolysis of carbohydrate polymers to fermentable sugars, and fermentation of these sugars to ethanol. Consolidated bioprocessing (CBP) approaches combine enzyme production, substrate hydrolysis, and fermentation into one process. Despite continued reductions in the cost of hydrolytic enzymes, CBP remains an attractive alternative due to the potential of decreasing costs associated with reducing unit operations and exogenous enzyme supplementation [12-14].

*Clostridium thermocellum* is a thermophilic and cellulolytic Gram-positive bacterium that is considered a consolidated bioprocessing candidate because of its ability to grow rapidly on native crystalline cellulose without the addition of exogenous enzymes. Moreover, *C. thermocellum* fermentations showed the utilization of up to 75% of thecellulose contained in pretreated biomass sources [7,15-17]. However, the wild-type strain has a mixed-product fermentation profile consisting of lactic acid, formic acid, acetic acid, and ethanol as well as carbon dioxide and hydrogen. As a result strain engineering approaches are needed to increase the yield of ethanol and minimize the production of the other metabolites [18]. Argyros *et al.*[19] developed a strain of *C. thermocellum* (M1570) that was evolved for growth and contained deletions in the acetic and lactic acid pathways (Δhpt Δldh Δpta). The strain produced ethanol in a 40:1 ratio with respect to residual organic acids in actively pH controlled Avicel fermentations.

Here we expand on the previous work of Fu *et al.*[7] and Yee *et al.*[8] where they showed that downregulation of the caffeic acid 3-*O-*methyltransferase (COMT) gene in switchgrass reduced recalcitrance and improved microbial bioconversion yield regardless of pretreatment condition for simultaneous saccharification and fermentation (SSF) with *S. cerevisiae*[7,8]. In contrast to yeast-based SSF, *C. thermocellum* (ATCC 27405) fermentations demonstrated sensitivity to the COMT transgenic biomass. It was postulated that additional soluble lignin pathway-derived constituents resulting from the COMT gene disruption measured by GC-MS in the fermentation broth may have led to the sensitivity and/or inhibition [8,20]. The fermentation sensitivity was not observed in the fermentation of the wild-type switchgrass line. This inhibition can be eliminated by extensive washing or hot water extraction, which allowed *C. thermocellum* to have a superior yield of fermentation products with the transgenic substrate versus wild-type line biomass, exceeding the yeast-based SSF yield [7,8]. In this study we fermented the less recalcitrant transgenic COMT switchgrass with the recombinant *C. thermocellum* strain M1570 developed by Argyros *et al.*[19]. We show that the fermentation of the transgenic biomass had superior conversion in comparison to the wild-type switchgrass and the primary fermentation end-product metabolite is ethanol at a 9:1 ratio of ethanol to acetic acid.

## Results

From the previous studies, fermentation of dilute-acid pretreated and extensively washed switchgrass had higher conversions than hot water or no pretreatment [7,8]. As a result the biomass was dilute acid pretreated and hot water extracted prior to fermentation. The carbohydrate composition of the biomass after dilute acid pretreatment was primarily glucan from cellulose measured by the quantitative saccharification assay. The dilute acid pretreatment solubilized the hemicellulose and the extensive washing removed soluble compounds and extractives.

**Figure 1:**
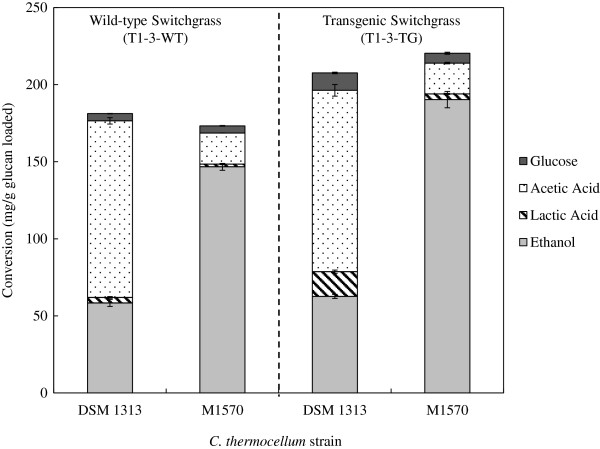
**Conversion (mg/g glucan loaded) for*C. thermocellum*mutant M1570 and wild-type DSM 1313 strains on both transgenic (T1-3-TG) and wild-type (T1-3-WT) switchgrass.** The standard deviation is from the average of triplicate buffered serum bottle fermentations. The light gray bars represent ethanol, the dashed bar represents lactic acid, the dotted bar represents acetic acid, and the dark gray bar represents soluble unfermented glucose.

The mutant (M1570) and wild-type (DSM 1313) *C. thermocellum* fermentations of the pretreated and washed transgenic (T1-3-TG) and wild-type control (T1-3-WT) switchgrass lines were monitored over time by tracking weight loss to gaseous products through periodic venting of the tared serum bottle. At 145 hours the weight loss stabilized indicating that the fermentations had ceased. Analysis of the endpoint fermentation broth was performed using HPLC measuring soluble sugars (glucose, cellobiose, arabinose, and xylose) and the primary fermentation metabolites for *C. thermocellum* (acetic acid, lactic acid, ethanol, and formic acid). The fermentation broths did not contain formic acid, cellobiose, arabinose, or xylose as measured by HPLC. The endpoint conversion was reported as mg/g glucan loaded for both metabolites and soluble but unfermented glucose. The transgenic line had greater conversion to fermentation metabolites than the wild-type by 11 and 27% for DSM 1313 and M1570, respectively (Figure 1, Table 1, and Additional file 1: Table S1). Moreover, the product profiles showed that ethanol was approximately 90% of the total metabolite conversion for fermentations with M1570 on either the transgenic or the wild-type switchgrass (1.7 g/L and 1.2 g/L, respectively). In contrast, the DSM 1313 fermentations produced acetic acid and ethanol at a ratio of approximately 2:1 for both transgenic and wild-type switchgrass with minimal amounts of lactic acid production.

**Table 1:** **Endpoint fermentation analysis of** ***C. thermocellum*** **strains on transgenic and wild-type switchgrass**

**Strain/Switchgrass**	**Total products (acetic acid + lactic acid + ethanol) (mg)**	**Glucan liberated from biomass (mg)**	**Yield (mg total products/g glucan liberated)**	**Conversion (mg total products/g glucan loaded)**
DSM 1313/T1-3-WT	73.1 ± 2.0	226 ± 5	324 ± 6	178 ± 2
DSM 1313/T1-3-TG	87.3 ± 3.0	264 ± 16	332 ± 26	198 ± 6
M1570/T1-3-WT	69.5 ± 1.2	232 ± 13	300 ± 17	170 ± 3
M1570/T1-3-TG	95.8 ± 2.6	309 ± 13	310 ± 16	216 ± 6

The T1-3-TG fermentation with M1570 had a conversion of 0.19 g/g glucan loaded or a yield of 0.27 g/g glucan liberated (Additional file 1: Table S1 and Additional file 2: Table S2), which is equivalent to the 0.27 g/g yield of ethanol achieved in the pH controlled Avicel fermentations by Argyros *et al.*[19]. The mutant and the wild-type *C. thermocellum* strains had similar conversions for fermentations on the same biomass line but significantly different metabolite ratios (Figure 1 and Additional file 1: Table S1). There were not substantial concentrations of liberated but unfermented sugars in the broth in the M1570 strain fermentations, but there was a slight increase of a 2.4-fold in residual, soluble, unfermented glucose in the DSM 1313 strain fermentation of the T1-3-TG in comparison to the T1-3-WT (Figure 1 and Additional file 1: Table S1). This could be due to the fact that these fermentations were heavily buffered to pH 7.0, but not actively pH controlled while the optimal growth range is above 6.2. The endpoint pH was approximately 6.3 for the M1570 fermentations and approximately 5.8 for the wild type *C. thermocellum* fermentations which is below the optimal growth range. Previous results showed an 18% improvement for fermentations with wild-type *C. thermocellum* (ATCC 27405) in actively pH controlled fermentations on COMT switchgrass [7]. Here we only show an 11% improvement in a buffered batch system; the residual, soluble, unfermented glucose is reported and cannot account for this difference. The pH and headspace effects may account for this difference in conversion of the transgenic biomass compared to the wild-type line. In addition to conversion, yield (mg/g glucan liberated) was evaluated for the fermentations described above. The glucan liberated from the biomass was determined by analysis of the solid fermentation residues using a quantitative saccharification assay for carbohydrate composition. The yield was calculated based on the assumption that all glucan liberated was converted to fermentation metabolites measured by HPLC (Table 1). A change in microbe from the wild-type (DSM 1313) to the mutant (M1570) significantly changed the product ratio from approximately 2:1 to 1:9 acetic acid to ethanol, regardless of wild-type versus transgenic switchgrass, but not the overall yield. A change from wild-type to transgenic switchgrass, improved the accessibility and conversion of the glucan, regardless of microbe, but not the overall fermentation yield. Moreover, regardless of the *C. thermocellum* strain, the transgenic switchgrass had more glucan liberated indicating that the cellulose was more easily accessible. Analysis of endpoint fermentation metabolites and unfermented soluble glucose indicated a total mass balance closure of approximately 60% for the above fermentations. This calculation was based on a stoichiometric ratio of 1:1 for ethanol to CO_2_ and acetic acid to CO_2_. In addition, it does not include maintenance, cell density, and protein production. The mass balance remains open and that calculation coupled with low yields suggests that carbon may be diverted through alternate pathways to side products that were not measured.

## Discussion

Transgenic feedstocks with reduced recalcitrance in combination with consolidated bioprocessing microbes have the potential to further reduce the cost of biofuels by eliminating or drastically reducing the requirement of exogenous hydrolytic enzymes. Therefore we examined the fermentation performance of the *C. thermocellum* M1570 strain on transgenic COMT switchgrass. We showed that fermentations with either the wild-type or mutant *C. thermocellum* strain on the transgenic switchgrass had improved conversion (mg/g glucan loaded), total products, and glucose liberated in comparison to the wild-type switchgrass indicating that the transgenic switchgrass was more easily digested. Moreover, the conversion for the M1570 strain was 90% ethanol with minimal amounts of the side products lactic and acetic acid regardless of switchgrass line. Fermentations of transgenic or wild-type switchgrass by either *C. thermocellum* strain gave comparable total end-product fermentation yields (mg/g glucan liberated), however with significantly different metabolite ratios. The similar yields and minimal residual unfermented carbohydrates in the fermentation broth indicate that the glucan liberated and utilized for measured endpoint fermentation products was similar for both strains.

The mass balance remains open for these fermentations where only endpoint metabolites and soluble but unfermented glucose is considered. The mass balance closure was approximately 60% under the assumption that glucan liberated was converted to fermentation metabolites and CO_2_ was estimated using the stoichiometric ratio of 1:1 for both ethanol to CO_2_ and acetic acid to CO_2_. In addition, both *C. thermocellum* strains had low endpoint fermentation yields and this may be in part be due to carbon flow through alternate pathways. Argyros *et al.*[19] and van der Veen *et al.*[21] both hypothesized that the disruption of fermentation metabolic pathways can lead to a redox imbalance and metabolism bottlenecks. They both showed that there is an accumulation of central metabolites, such as pyruvate, in their deletion strains. Moreover, van der Veen *et al.*[21] showed that their evolved Δhpt Δldh Δpta mutant channeled 17% of the available carbon to secreted amino acids. The open mass balance and low yields from these fermentations suggests that there is a redox-imbalance in Δhpt Δpta Δldh mutant strain and the carbon is diverted through alternate pathways. This issue may be potentially solved through further study of intracellular redox state and metabolic pathways leading to alternate engineering strategies [22-24].

## Conclusions

This set of fermentations establishes a baseline for CBP conversion of transgenic switchgrass with a genetically engineered and evolved *C. thermocellum* strain (M1570) that produces primarily ethanol. The mutant strain responded to the reduced recalcitrant transgenic COMT switchgrass with the same trend as the wild-type *C. thermocellum*. The ethanol yield for the mutant strain was significantly higher for the fermentation of the transgenic versus the wild-type control switchgrass. These results give additional support toward the potential of transgenic COMT switchgrass as a biomass feedstock line with reduced recalcitrance and improved fermentation yield. With further understanding of intracellular metabolism and redox state leading to engineered strains with improved ethanol yield, rate, and titer, CBP with *C. thermocellum* mutants in combination with biomass with reduced recalcitrance have the potential to further reduce the costs of cellulosic ethanol.

## Materials and methods

### Growth and harvesting conditions for transgenic and control plant material

COMT down-regulated transgenic and wild-type control switchgrass (*Panicum virgatum L.*) lines were generated by the Samuel Roberts Noble Foundation as previously described in Fu *et al.*[7].

### Pretreatment

The biomass was milled in a Wiley mill using a 20 mesh screen (Thomas Scientific, Swedesboro, NJ, USA). Dilute acid pretreatment was performed as described previously in Fu *et al.*[7] and Yee *et al.*[8]. Briefly, the biomass was soaked overnight in 0.5% H_2_SO_4_ and loaded at a ratio of 2.5 g dry per Hastelloy steel tubular pretreatment reactor (Industrial Alloys Plus Inc., Utica, KY, USA). The reactors were preheated in boiling water for 2 minutes and then transferred to a fluidized sand bath (Omega FSB1:Techne Co., Cole Parmer, Court Vernon Hills, IL, USA) at 180°C for 7.5 minutes [7,25]. The reactors were cooled by quenching in an ice bath. The biomass was washed with 100 mL Milli-Q water (Millipore Corporation, Billerica, MA, USA) per gram dry biomass and then subjected to a hot water extraction to remove inhibitory water soluble compounds and washed a second time as described previously by Yee *et al.*[8]. The solid residual biomass was stored at −20°C until fermentation.

### Consolidated bioprocessing conversion

The Lee R. Lynd Lab at Dartmouth College (Hanover, NH, USA) generously donated the *C. thermocellum* DSM 1313 strain and the Mascoma Corporation (Lebanon, NH, USA) provided the *C. thermocellum* M1570 strain. The M1570 strain was developed in the *C. thermocellum* DSM 1313 Δhpt background strain. Lactate dehydrogenase (Ldh) and phosphotransacetylase (Pta) genes were deleted and the Δhpt Δldh Δpta mutant strain was transferred in batch culture for 2,000 hours and a stable strain was achieved [19]. All CBP fermentations were cultivated at 55°C and underwent orbital shaking at 125 rpm in defined no-yeast extract media for thermophilic clostridia (MTC) [26]. Bottles were loaded with 0.75 g of biomass on a dry basis, autoclaved for 30 minutes for sterilization purposes, and a 2.0% vol/vol inoculum was used for a final volume of 50 mL. The inoculum was grown in 125 mL anaerobic serum bottles with 50 mL of MTC media and a carbon source of 5.0 g/L Avicel (FMC BioPolymer, Newark, DE, USA) at 125 rpm and 55°C. Samples were not removed from the serum bottles during fermentation; instead weight loss was used to monitor the progress of the fermentation [7,8,10,27].

### Analytical methods

Endpoint fermentation broth samples were analyzed for metabolites (acetic acid, lactic acid, and ethanol) and residual carbohydrates (cellobiose, glucose, xylose, arabinose) using a high performance liquid chromatography (HPLC) LaChrom Elite™ system (Hitachi High Technologies America Inc., Schaumburg, IL, USA) equipped with a refractive index detector (model L-2490). The products and carbohydrates were separated using an Aminex™ HPX-87H column (Bio-Rad Laboratories Inc., Hercules, CA, USA) at a flow rate of 0.5 mL/min of 5.0 mM sulfuric acid and a column temperature of 60°C [7,8,16].

Pretreated and washed biomass and fermentation residues were analyzed for carbohydrate composition using a quantitative saccharification assay NREL/TP-510-42618 and HPLC method NREL/TP-510-42623. Briefly, the samples were analyzed for carbohydrate composition using a high performance liquid chromatography (HPLC) LaChrom Elite™ system (Hitachi High Technologies America Inc.) equipped with a refractive index detector (model L-2490) and a UV-Vis detector (model L-2420). The carbohydrates (glucose, xylose, galactose, mannose, and arabinose) and pentose and hexose sugar degradation products (furfural and 5-hydroxy methyl furfural) were separated using an Aminex™ HPX-87P column (Bio-Rad Laboratories Inc.), at a 0.6 mL/min flow rate of water and a column temperature of 80°C [7,8].

*C. thermocellum* utilizes hexose sugars, and after dilute acid pretreatment and extensive washing the hexose sugar content of the biomass was primarily glucan from cellulose because the dilute acid pretreatment solubilized the hemicellulose and the extensive washing removed extractives and soluble components. Analysis of the carbohydrate composition post-pretreatment and washing showed negligible amounts of mannose, arabinose, and xylose. Both prior to and post-fermentation the carbohydrate content of the biomass was determined by the quantitative saccharification assay. The conversion was calculated based on the initial glucan content of the biomass loaded and the yield was calculated based on glucan liberated from the biomass. Both conversion and yield are calculated under the assumption that all available glucan was converted to fermentation products.

## Abbreviations

ATCC: American type culture collection; CBP: Consolidated bioprocessing; COMT: Caffeic acid 3-O-methyltransferase; CTRL: Control; DA: Dilute acid pretreatment; HPLC: High performance liquid chromatography; HW: Hot water; SSF: Simultaneous saccharification and fermentation; T1: Generation one; TG: Transgenic; WT: Wild-type.

## Competing interests

The authors declare that they have no competing interests.

## Authors’ contributions

KLY planned the work, conducted the experiments and wrote the manuscript. MRJr assisted in data acquisition and analysis and edited the manuscript. OAT helped run the fermentations and edited the manuscript. CF and ZYW produced and supplied the transgenic switchgrass material and edited the manuscript. JRM helped plan the experiments and edited the manuscript. BHD helped interpret the results and plan and edit the manuscript. All authors have read and approved the final manuscript.

## Supplementary Material

Additional file 1:Table S1 Endpoint conversion (mg/g glucan loaded) for products and soluble unfermented glucose. Click here for file

Additional file 2:Table S2 Endpoint yield (mg/g glucose liberated) for products and soluble unfermented glucose. Click here for file
