# Supplementary material for: Consolidated bioprocessing of transgenic switchgrass by an engineered and evolved Clostridium thermocellum strain
Source: Biotechnol Biofuels. 2014 May 22;7:75. doi: 10.1186/1754-6834-7-75 (PMC4037551; doi:10.1186/1754-6834-7-75)
Supplement: Additional file 1:Table S1 — Endpoint conversion (mg/g glucan loaded) for products and soluble unfermented glucose. [file 1754-6834-7-75-S1.docx]

**Additional file 1: Table S1.**

| Strain / Switchgrass | Ethanol (mg/g initial glucan) | Lactic Acid (mg/g initial glucan) | Acetic Acid (mg/g initial glucan) | Glucose (mg/g initial glucan) |
| --- | --- | --- | --- | --- |
| DSM 1313 / T1-3-WT | 58.4±2.2 | 3.6±0.6 | 114.6±2.1 | 4.7±0.1 |
| DSM 1313 / T1-3-TG | 62.6±1.3 | 16.0±1.0 | 117.7±3.8 | 11.3±0.2 |
| M1570 / T1-3-WT | 146.7±2.2 | 1.8±0.4 | 20.2±0.1 | 4.6±0.5 |
| M1570 / T1-3-TG | 190.3±5.3 | 3.7±0.4 | 20.0±0.5 | 6.4±0.7 |
