# Supplementary material for: Consolidated bioprocessing of transgenic switchgrass by an engineered and evolved Clostridium thermocellum strain
Source: Biotechnol Biofuels. 2014 May 22;7:75. doi: 10.1186/1754-6834-7-75 (PMC4037551; doi:10.1186/1754-6834-7-75)
Supplement: Additional file 2:Table S2 — Endpoint yield (mg/g glucose liberated) for products and soluble unfermented glucose. [file 1754-6834-7-75-S2.docx]

**Additional file 2: Table S2.**

| Strain / Biomass | Ethanol (mg/g glucan liberated) | Lactate (mg/g glucan liberated) | Acetate (mg/g glucan liberated) | Glucose (mg/g glucan liberated) |
| --- | --- | --- | --- | --- |
| DSM 1313 / T1-3-WT | 106.9±3.9 | 6.6±1.0 | 212.8±4.2 | 8.6±0.3 |
| DSM 1313 / T1-3-TG | 119.1±2.9 | 30.5±2.1 | 226.6±7.9 | 18.9±4.6 |
| M1570 / T1-3-WT | 229.8±3.8 | 2.7±0.6 | 31.2±0.2 | 7.2±0.8 |
| M1570 / T1-3-TG | 275.8±7.1 | 5.3±0.6 | 28.8±0.7 | 9.2±0.9 |
